# Perceived Impact of the COVID-19 Lockdown on the Family Context of Foster and Non-Foster Families

**DOI:** 10.1007/s10826-021-02185-x

**Published:** 2021-12-06

**Authors:** Lucía González-Pasarín, Antonio Urbano-Contreras, Isabel M. Bernedo, Jesús Oliver

**Affiliations:** 1 Departamento de Psicología Evolutiva y de la Educación, Facultad de Psicología, Universidad de Málaga, Campus de Teatinos S/N, 29071 Málaga, Spain; 2 Departamento de Ciencias de la Educación, Facultad de Formación del Profesorado y Educación, Universidad de Oviedo, C/ Aniceto Sela, 1, 33005 Oviedo, Spain; 3 Departamento de Psicología, Facultad de Ciencias Humanas y Sociales, Universidad Pontificia Comillas, Universidad de Comillas, 3-5, 28049 Madrid, Spain

**Keywords:** Adaptability, Cohesion, Stress, Family context, Foster families

## Abstract

The COVID-19 pandemic and the resulting lockdown have had a far-reaching impact across all levels of society. In Spain, severe restrictions were placed on people’s mobility, and leaving the home was only possible under special circumstances. This study analyzes the impact of lockdown on the family context of foster and non-foster families, focusing particularly on their levels of cohesion, adaptability, and perceived stress. It also examines a series of variables that may have influenced foster families’ perceptions of their family context during lockdown. Data were gathered through an online survey that was completed by 347 individuals corresponding to 100 foster families and 247 non-foster families from different regions of Spain. Analyses were descriptive and exploratory in nature. The results appear to suggest that lockdown has had a greater impact on the family context of non-foster families. With respect to foster families’ experiences of lockdown, variables such as loss of employment and having a child with special educational needs would seem to be important. For both types of families, lockdown has provided an opportunity to improve certain aspects of their family context. Given that further lockdowns of some degree may be necessary in the future, it is important to ensure that families have access to the psychoeducational resources they need to maintain, as far as possible, a positive family context.

## Highlights

- We examined the impact of the COVID-19 lockdown on foster and non-foster families.
- Family context was perceived to be better under lockdown than previously.
- Lockdown had a greater impact on the family context of non-foster families.
- Foster family perceptions were influenced by unemployment.
- Having a child with special educational needs was also a factor in foster families.

Although it remains to be seen what long-term effects the COVID-19 pandemic will have on societies across the world, it is already clear that lockdown has had a major socioeconomic impact. Health systems have faced unprecedented strain due to the high number of infections, and coupled with the significant loss of life this has exposed frontline staff to enormous levels of stress, with the inevitable psychological consequences (Moya et al., 2020). In terms of the economy and employment, an already fragile situation has been made even worse by the pandemic. As noted by Moya et al. (2020), the IMF is forecasting dire economic consequences, especially as regards unemployment. In Spain, where the present study was conducted, it is estimated that during the first two months of lockdown in 2020, 947,896 jobs were lost and 3.4 million employees were furloughed, with unemployment reaching levels not seen since May 2016 (EFE News Agency, May 5, 2020).

Because the situation is constantly changing in response to the evolution and effects of the pandemic, the general climate is one of uncertainty, which makes coping even more challenging. This uncertainty extends not only to future employment prospects but also to the future of family and interpersonal relationships, thus placing considerable stress on individuals, families, and society as a whole. With specific regard to the psychological and emotional consequences of the crisis, researchers have already posited an increased risk of family violence as a result of the COVID-19 pandemic (Campbell, 2020; Usher et al., 2020).

From the perspective of Bronfenbrenner’s ecological (Bronfenbrenner, 1979) and bioecological model (Bronfenbrenner and Morris, 2006), and its latest development in the form of the Process–Person–Context–Time model (Bronfenbrenner and Evans (2000); Bronfenbrenner and Morris, 2006), the COVID-19 pandemic and, especially, the associated lockdown may be considered a social phenomenon that is affecting all individuals in all their life contexts, and hence one target of analysis is its impact on the functioning of the family microsystem. According to the aforementioned model, we are immersed from birth in a set of nested systems which exert bidirectional and transactional influences of a social, cultural, and historical nature, systems which themselves are subject to processes of change (Bronfenbrenner, 1979; Bronfenbrenner and Ceci, 1994). The model distinguishes four systems of influence: microsystem (family, school, friends, and work), mesosystem (interrelationships between the microsystems), exosystem (extended family, social institutions, and the mass media), and macrosystem (social values and sociocultural norms) (Bronfenbrenner, 1979). The current crisis due to COVID-19 is a macrosystem event that affects the other levels of influence in the context of a constantly evolving globalized world.

For children, the primary developmental context is the family, which must provide a secure affective base so as to ensure the child’s survival, growth, and socialization (Bowlby, 1985, 2014; López, 2009). Families must therefore have skills and competences that help to foster adequate psychological adjustment in children (Alarcón, 2017; Bisquerra et al., 2015), and family functioning will depend to a large extent on these skills. The importance of the family in this respect is acknowledged in the Council of Europe’s (2006) Recommendation (Rec2006/19) on Policy to Support Positive Parenting, which urges member states to provide families with the psychoeducational support they need in order to fulfill their responsibilities in relation to children’s upbringing, care, and education. This implies a combined effort across the spheres of education, social services, and health, backed where necessary by policy and legislation.

In studying the family context and functioning, two key relational aspects to consider are cohesion and adaptability (Urbano et al., 2018; Zuñeda et al., 2016). Importantly, various studies have linked family cohesion and adaptability to better psychological adjustment in children, adolescents, and adults, as well as to family satisfaction (Olson, 2011; Prioste et al., 2020; Rivero et al., 2010).

Despite the importance of the family system for children’s psychosocial development, there are occasions when a family’s circumstances make it unadvisable for a child to remain with his or her birth parents. In such cases, one of the options that will be considered by child protection agencies is family foster care. If a child is considered to be at risk, then fostering offers greater protection than would a family support program, while remaining a less drastic measure than adoption, which implies a permanent separation from the birth family (Urbano and Bernedo, 2016).

Research to date has not specifically examined cohesion and adaptability in foster families, with studies tending to focus on identifying the factors that may influence placement outcomes and the foster child’s wellbeing, for example, family stress (Fuentes et al., 2015; Julien-Chinn et al., 2017; Salas et al., 2015, 2016). Generally speaking, the impact that stressors ultimately have on a family depends on two aspects: the pile-up of stressors and the family’s resources and capabilities for dealing with them. When the pile-up of demands overwhelms a family’s resources, then it is highly likely that children’s wellbeing will be affected (Lorence et al., 2009, 2013).

Although the primary objective of foster care is to protect the child, foster families in fact have highly varied and complex functions, the specifics of which will be determined by a particular child’s needs and any problems resulting from his or her personal history. Dealing with these challenges can produce burden and stress in foster carers, which will then impact negatively on the placement, the foster family (Farmer et al., 2005; Oosterman et al., 2007), and the child, who as a result will be more likely to present behavior problems and difficulties with attention and impulse control (Bernedo et al., 2012, 2014; Palacios et al., 2014; Salas et al., 2015, 2016). Importantly, the level of stress experienced by foster carers has also been linked to their choice of parenting style. Thus, lower levels of stress are associated with more democratic parenting styles, which in turn are associated, in foster children, with less frequent aggressive behavior and negative mood states, better social skills, and a stronger affective bond with the foster carers (Fernandez, 2009; Jiménez and Palacios, 2009). Conversely, permissive or authoritarian parenting styles are related to greater stress in foster carers and more emotional and behavioral problems in children (Jiménez and Palacios, 2009; Salas et al., 2015, 2016). What research in this field has rarely explored, however, are possible differences between foster and non-foster families in relation to aspects such as parenting style. One exception to this is a study conducted in Spain by Bernedo et al. (2007) that examined perceptions of parental socialization styles in adoptive and non-adoptive families. The authors found that from the point of view of both adolescents and parents, adoptive families were perceived as being more democratic (i.e., they showed greater warmth and were more communicative and inductive, and less critical and indulgent) than were non-adoptive families (Bernedo et al. 2007).

The economic and social instability brought about by the COVID-19 pandemic is obviously an additional source of stress for families, especially those who have experienced loss of employment and who are now struggling to cover their basic needs. For these families, the availability of financial and material support can make an important contribution to their emotional wellbeing (Estévez, 2016). It is important to note that many Spanish families will have been affected in this way, insofar as the early months of lockdown saw significant job losses (EFE News Agency, May 5, 2020) in the arts and entertainment industry (20,702 jobs lost), the hospitality sector (76,902), and in construction (89,864). Only in the health and social services sector did employment rise (23,228 more employees, as would be expected during a health crisis).

In light of the above, the main aim of this descriptive and exploratory study was to analyze the impact of the COVID-19 lockdown on the family context of both foster and non-foster families, focusing particularly on their levels of cohesion, adaptability (family functioning), and perceived stress. We also examine a series of variables that may have influenced foster families’ perceptions of their family context during lockdown: loss of employment as a result of the COVID-19 pandemic, having a relative or close friend who contracted COVID-19, and having a child with special educational needs at home. The results should provide insight into those aspects of family functioning where foster and non-foster families need support (e.g., communication and conflict resolution skills or measures to reduce family stress).

## Method

### Participants

The sample comprised 347 Spanish adults (81.3% female) corresponding to 100 foster families and 247 non-foster families. They came from different regions of Spain and had a mean age of 45.6 years (SD = 8.1). The majority of participants had secondary or higher education (83%) and around two-thirds of the sample was currently employed (64%). Among those in work, 68.3% were working from home during lockdown. Table 1 summarizes the characteristics of the foster families and non-foster families.

**Table 1:** Sociodemographic characteristics of the sample

Variable	Foster families	Non-foster families
Age (years)^a^	48.6 (8.0)	44.4 (7.8)
Gender		
Female	83%	80.6%
Male	17%	19.4%
Spanish nationality	94%	92.7%
No. of foster and/or biological children in the household^a^	2.2 (1.2)	1.7 (0.7)
Child with special educational needs	24%	4.9%
Monthly family income		
<€1000	7%	6%
€1001–1500	21%	13.8%
€1501–2000	29%	18.2%
€2001–2500	21%	21.5%
>€2500	22%	40.5%
Relative or close friend contracted COVID-19	36%	42.5%
Loss of employment during lockdown	33%	66%
Kind of foster care		
Emergency	28%	-
Short-term	13%	-
Long-term	59%	-

^a^Mean, standard deviation in parentheses

Participants from foster families were older (*t*(345) = −4.50, *p* < 0.001, *d* = 0.53), had more children (*t*(345) = −2.08, *p* = 0.038, *d* = 0.25), and more children with special needs (*t*(345) = −4.25, *p* < 0.001, *d* = 0.65) than did participants from non-foster families. In addition, a smaller percentage of foster families had a monthly family income over €2500 (*χ*^*2*^(5) = 13.54, *p* = 0.019, *C* = 0.19).

### Instruments

Participants completed an online survey comprising two scales and a sociodemographic questionnaire, specifically:

#### Family Adaptability and Cohesion Evaluation Scale-20Esp (FACES-20Esp)

The FACES II was originally developed by Olson et al. (1982) as a tool for evaluating family functioning. Here we used the Spanish adaptation of this scale (Martínez-Pampliega et al., 2006), which comprises 20 items, each with five response options (from 1, *Never or hardly ever* to 5, *Always or almost always*) and distributed across two subscales, Cohesion and Adaptability (10 items each). For the present study, we added an extra item (“*In general, the atmosphere in my family is good*”) in order to obtain an overall measure and to study the concurrent validity of the instrument. The correlations between this item and the other 20 items were significant (p ≤ 0.00, two-tailed). Cronbach’s alpha coefficients were 0.89 for cohesion, 0.87 for adaptability, and 0.95 for the total score. These values were the same for perceived family functioning before and during lockdown. When pooling the two measures (i.e., ratings for before and during lockdown), alpha increased to 0.97, which may be considered excellent (George and Mallery, 2003). In the present study we used the total score as a single measure of family functioning because factor analyses carried out by Martínez-Pampliega et al. (2006, 2011) suggested that the Spanish version may be considered unidimensional.

#### Family Stress Scale—Spanish Version

We assessed the level of family stress using the Spanish version (Sanz et al., 2002) of the Family Stress Scale (Olson et al. 1982), which comprises 20 items, each with five response options (from 1, *Never or hardly ever* to 5, *Very often*). In order to reduce fatigue among participants, we only used items 1, 4, 6, 7, 8, 9, 10, 16, and 18 of this scale, with item 10 being reformulated as “Burden related to children’s school work”. Internal consistency of scores for perceived family stress before and during lockdown was 0.70 and 0.72, respectively. The alpha coefficient when pooling the two measures was 0.83, indicating good reliability (George and Mallery, 2003).

#### Sociodemographic questionnaire

This questionnaire was developed ad hoc for the present study to gather sociodemographic data (e.g., gender, age, employment status, level of education, children, monthly family income) and information about the impact of COVID-19 on the family (whether any members of the household, other relatives, or friends had developed symptoms; whether a family member or friend had lost their job).

### Procedure

In this cross-sectional study, the aforementioned survey was hosted online in Google Forms and respondents were asked to rate their family situation both before and during lockdown. Data were collected over a seven-week period from March 30 to May 17, 2020.

Participants were recruited by means of convenience snowball sampling, with an invitation to complete the questionnaire being sent to various institutions from different regions of Spain, namely universities (e.g., Malaga, Oviedo, and Pontificia Comillas) and associations and organizations with links to the area of family fostering (e.g., Infania, ASEAF, Aldaima, Apraf-a, Acógeles).

It was made clear that participation was voluntary and that all data would remain anonymous throughout. The first item of the online survey requested participants’ consent to use the information they provided for research purposes, and without an affirmative response the software would not allow them to continue.

### Data Analysis

Data were analyzed using SPSS 25.0 (IBM Corp., 2017). We carried out descriptive and frequency analyses to determine the characteristics of foster and non-foster families, using the Student’s *t* test for two independent samples to examine differences between the two groups. In order to analyze the impact of lockdown on the family context (i.e., comparison of perceived family dynamics before vs. during lockdown in each group) we applied the Student’s *t* test for related samples. Cohen’s *d* was calculated as a measure of effect size.

## Results

We begin by presenting the results for the impact of lockdown on perceived cohesion, adaptability, and stress in foster and non-foster families. Next, we describe the results obtained when comparing the two groups of families on the variables studied. Finally, we consider the relationship between the variables studied and certain characteristics of foster families.

### Adaptability and Cohesion in Foster and Non-Foster Families

In terms of adaptability and cohesion (Table 2), lockdown appears to have had a greater impact on non-foster families (statistically significant differences on 12 FACES items) than foster families (significant differences on 5 items). The direction of the differences is the same, however, and although the associated effect sizes are generally small, both types of families perceived an improvement in family functioning during lockdown. The same pattern was observed for total scores on the FACES, which were also significantly higher during (vs. before) lockdown in both types of families, although the difference was greater among non-foster families.

**Table 2:** Family adaptability and cohesion before and during lockdown

Items	Foster family	Before	During	*p* (*d*)
		*M* (*SD*)	*M* (*SD*)	
1. We feel very close to each other	Yes	4.46 (0.79)	4.58 (0.69)	0.109
	No	4.16 (0.89)	4.37 (0.80)	0.000 (0.25)
2. When there are problems to be dealt with, we follow our children’s suggestions	Yes	3.44 (0.86)	3.78 (0.89)	0.000 (0.39)
	No	3.10 (0.94)	3.48 (1.02)	0.000 (0.39)
3. Discipline (rules, obligations, punishments) is fair	Yes	3.89 (0.84)	3.97 (0.91)	0.296
	No	3.88 (0.90)	3.89 (0.88)	0.792
4. We accept the decisions we make as a family	Yes	4.10 (0.90)	4.11 (0.80)	0.889
	No	4.04 (0.91)	4.15 (0.86)	0.031 (0.12)
5. When it comes to discipline, we take our children’s opinion into account	Yes	3.78 (0.88)	3.82 (0.95)	0.596
	No	3.43 (1.04)	3.56 (1.05)	0.015 (0.12)
6. When problems arise, we negotiate to find a solution	Yes	4.03 (0.88)	4.14 (0.88)	0.220
	No	3.96 (0.93)	4.05 (0.88)	0.089
7. We do things together	Yes	4.06 (0.87)	4.17 (0.89)	0.167
	No	4.09 (0.89)	4.21 (0.82)	0.014 (0.14)
8. We freely decide what we want to do	Yes	3.96 (0.92)	4.07 (0.94)	0.101
	No	3.91 (0.93)	4.03 (0.92)	0.031 (0.13)
9. We get together in the same room	Yes	4.30 (0.73)	4.41 (0.79)	0.105
	No	4.27 (0.87)	4.38 (0.79)	0.022 (0.13)
10. We support each other during difficult times	Yes	4.64 (0.73)	4.67 (0.71)	0.494
	No	4.49 (0.79)	4.51 (0.76)	0.630
11. Parents and children discuss punishment	Yes	4.28 (0.88)	4.09 (0.98)	0.021 (0.20)
	No	4.04 (1.08)	4.01 (1.02)	0.548
12. We all find it easy to express our opinion	Yes	3.84 (1.05)	3.90 (1.07)	0.525
	No	3.98 (0.98)	4.01 (0.88)	0.602
13. We share interests and hobbies	Yes	3.75 (0.91)	3.91 (0.85)	0.039 (0.18)
	No	3.72 (0.97)	3.88 (0.89)	0.001 (0.17)
14. We try new ways of dealing with problems	Yes	3.97 (0.91)	4.03 (0.94)	0.417
	No	3.80 (0.94)	3.90 (0.88)	0.029 (0.11)
15. We like to spend our free time together	Yes	4.14 (0.87)	4.23 (0.84)	0.161
	No	4.10 (0.94)	4.13 (0.92)	0.597
16. We all have a say in important family decisions	Yes	4.01 (0.95)	4.01 (1.01)	1.000
	No	3.92 (0.93)	3.96 (1.00)	0.392
17. We consult each other on personal decisions	Yes	4.13 (0.86)	4.19 (0.90)	0.306
	No	3.98 (0.95)	4.06 (0.88)	0.094
18. We ask each other for help	Yes	4.23 (0.89)	4.41 (0.87)	0.012 (0.20)
	No	4.18 (0.88)	4.28 (0.85)	0.027 (0.12)
19. We discuss problems and feel good about the solutions	Yes	3.95 (0.86)	4.09 (0.85)	0.043 (0.16)
	No	3.85 (0.92)	3.95 (0.85)	0.031 (0.11)
20. Family unity is important for us	Yes	4.21 (1.08)	4.33 (1.11)	0.086
	No	4.27 (0.92)	4.38 (0.90)	0.042 (0.12)
21. In general, the atmosphere in my family is good	Yes	4.28 (0.84)	4.36 (0.84)	0.158
	No	4.27 (0.81)	4.35 (0.77)	0.051
*Σ* FACES-20Esp (items 1–20)	Yes	81.48 (12.24)	82.88 (12.90)	0.042 (0.11)
	No	79.33 (12.95)	81.29 (12.14)	0.001 (0.16)

### Family Stress in Foster and Non-Foster Families

With respect to the level of family stress (Table 3), the results again suggest that the family context of non-foster families was more affected by lockdown (statistically significant differences on 4 items) than was the case among foster families (significant difference on 1 item). All these differences indicate a reduction in stress during lockdown, although once again the associated effect sizes are small. Note that when considering total scores on the Family Stress Scale, the perceived reduction in stress during lockdown was only statistically significant among non-foster families.

**Table 3:** Family stress before and during lockdown

Items	Foster family	Before	During	*p* (*d*)
		*M* (*SD*)	*M* (*SD*)	
22. Arguments between parents and children	Yes	3.13 (0.99)	2.94 (1.09)	0.083
	No	3.07 (1.05)	2.83 (1.04)	0.001 (0.23)
23. Physical illness or death of a family member	Yes	1.99 (1.26)	1.64 (1.10)	0.009 (0.30)
	No	1.90 (1.18)	1.55 (0.98)	0.000 (0.32)
24. Unresolved conflicts	Yes	1.77 (0.89)	1.84 (1.08)	0.485
	No	2.02 (1.07)	1.91 (1.00)	0.095
25. Difficulty paying the monthly bills	Yes	2.06 (1.16)	1.89 (1.16)	0.088
	No	1.82 (1.01)	1.81 (1.08)	0.814
26. Child care difficulties	Yes	1.80 (1.06)	1.85 (1.21)	0.686
	No	1.92 (1.11)	1.80 (1.04)	0.043 (0.11)
27. Emotional problems with family members (arguments, etc.)	Yes	2.05 (0.94)	2.09 (1.08)	0.702
	No	2.13 (1.04)	2.18 (0.95)	0.438
28. Burden related to children’s school work	Yes	2.69 (1.25)	2.80 (1.33)	0.305
	No	2.38 (1.16)	2.49 (1.34)	0.144
29. Problems sharing out household chores	Yes	2.35 (1.10)	2.38 (1.16)	0.716
	No	2.48 (1.13)	2.40 (1.12)	0.158
30. Lack of time to relax or switch off	Yes	2.84 (1.19)	2.71 (1.34)	0.276
	No	3.06 (1.16)	2.79 (1.28)	0.000 (0.22)
Σ Family Stress Scale	Yes	20.47 (5.44)	19.71 (6.01)	0.096
	No	20.83 (5.38)	19.83 (5.59)	0.003 (0.18)

### Family Context of Foster and Non-Foster Families

Regarding the family context, Table 4 shows that of the total number of variables analyzed (*n* = 30 items measuring family cohesion, adaptability, and stress), seven items yielded a significant difference between foster and non-foster families in their ratings for the period *before* lockdown. It can also be seen, however, that only three of these items (1, 2, and 5) continued to show a significant difference when participants were asked for their perceptions of the family context *during* lockdown. Analyzing how families felt prior to lockdown, foster families had a more positive view on all but two of these seven variables, the exceptions being that they reported more difficulty paying the monthly bills and a greater burden related to children’s school work. During lockdown, however, differences in perceptions of the family context between the two groups appear to diminish, and when differences are observed it is the foster families who report a more positive view. Comparison of foster and non-foster families based on their total scores on the two scales showed no statistically significant differences between the groups either before or during lockdown.

**Table 4:** Significant differences in the family context of foster and non-foster families before and/or during lockdown

Items	Time	Foster Families	Non-Foster Families	*p* (*d*)
		*M* (*SD*)	*M* (*SD*)	
1. We feel very close to each other	Before	4.47 (0.79)	4.16 (0.89)	0.002 (0.33)
	During	4.58 (0.69)	4.37 (0.80)	0.015 (0.34)
2. When there are problems to be dealt with, we follow our children’s suggestions	Before	3.43 (0.85)	3.10 (0.95)	0.003 (0.33)
	During	3.78 (0.89)	3.48 (1.02)	0.013 (0.27)
5. When it comes to discipline, we take our children’s opinion into account	Before	3.76 (0.91)	3.44 (1.04)	0.005 (0.39)
	During	3.82 (0.95)	3.56 (1.05)	0.039 (0.23)
11. Parents and children discuss punishment	Before	4.29 (0.87)	4.04 (1.07)	0.037 (0.23)
24. Unresolved conflicts	Before	1.74 (0.88)	2.02 (0.107)	0.020 (0.33)
25. Difficulty paying the monthly bills	Before	2.11 (1.20)	1.83 (1.02)	0.038 (0.33)
28. Burden related to children’s school work	Before	2.67 (1.28)	2.36 (1.16)	0.032 (0.23)

### Variables Associated with Perceptions of the Family Context in Foster Families

Finally, we analyzed a series of variables which, based on the literature, may have affected foster families’ perceptions of their family context during lockdown. Specifically, we examined the possible influence of: *loss of employment* as a result of the COVID-19 pandemic, having a *relative or close friend who contracted COVID-19*, and having a *child with special educational needs* at home.

With respect to loss of employment, and considering, first, foster families’ perceptions for the period before lockdown, a significant difference was only observed on item 26 (*Child care difficulties, p* = 0.030, *d* = 0.47), with those families who experienced loss of employment as a result of the pandemic reporting more difficulties (*M* = 1.91, *SD* = 1.15) than did those who remained in work (*M* = 1.48, *SD* = 0.76). When it came to their perceptions during lockdown, however, we observed differences on five items (see Table 5), and in each case, those foster families which had not been affected by loss of employment gave more positive ratings of their family context, especially in terms of their ability to adapt. These families also reported fewer financial difficulties and fewer problems sharing out the household chores.

**Table 5:** Significant differences in the family context of foster families according to loss of employment and having a child with special educational needs

Item	Unemployment			Special educational needs					
	During lockdown			Before lockdown			During lockdown		
	Yes	No	p (d)	Yes	No	p (d)	Yes	No	p (d)
2. When there are problems to be dealt with, we follow our children’s suggestions	3.65 (0.89)	4.06 (0.81)	0.030 (0.46)						
6. When problems arise, we negotiate to find a solution	4.00 (93)	4.42 (0.72)	0.029 (0.46)						
8. We freely decide what we want to do	3.92 (0.98)	4.39 (0.76)	0.022 (0.48)						
15. We like to spend our free time together							3.88 (1.04)	4.35 (0.74)	0.016 (0.51)
19. We discuss problems and feel good about the solutions				3.58 (0.83)	4.08 (0.83)	0.012 (0.52)			
21. In general, the atmosphere in my family is good				3.92 (1.02)	4.44 (0.72)	0.006 (0.57)	4.50 (0.93)	4.27 (1.17)	0.026 (0.47)
22. Arguments between parents and children							4.04 (1.08)	4.48 (0.71)	0.017 (0.66)
24. Unresolved conflicts							1.79 (1.26)	1.61 (1.07)	0.015 (0.52)
25. Difficulty paying the monthly bills	2.03 (1.26)	1.58 (0.85)	0.042 (0.45)						
26. Child care difficulties				2.17 (1.09)	1.64 (1.02)	0.034 (0.44)	2.29 (1.27)	1.71 (1.17)	0.041 (0.43)
27. Emotional problems with family members (arguments, etc.)							2.50 (1.18)	1.96 (1.01)	0.032 (0.45)
28. Burden related to children’s school work				3.26 (1.29)	2.49 (1.23)	0.010 (0.53)	3.38 (1.31)	2.63 (1.28)	0.015 (0.51)
29. Problems sharing out household chores	2.55 (1.20)	2.03 (1.02)	0.039 (0.43)				2.88 (1.23)	2.22 (1.10)	0.017 (0.50)

Values for item scores are mean (SD)

Regarding the family context of foster families where a relative or close friend had contracted COVID-19, the results for the period before lockdown revealed significant differences on items 20 (*Family unity is important for us*, *p* = 0.020, *d* = 0.48), 22 (*Arguments between parents and children*, *p* = 0.020, *d* = 0.46), and 24 (*Unresolved conflicts*, *p* = 0.049, *d* = 0.41), with scores in each case being higher among families in which a loved one had contracted COVID-19 (*M* = 4.54, *SD* = 0.66, *M* = 3.42, *SD* = 0.84, and *M* = 1.97, *SD* = 0.95 vs. *M* = 4.09, *SD* = 1.21, *M* = 2.92, *SD* = 1.08, and *M* = 1.61, *SD* = 0.81, respectively). However, only one of these items continued to be associated with a significant difference during lockdown (item 22, *Arguments between parents and children*, *p* = 0.016, *d* = 0.51), although a difference also emerged here on item 25 (*Difficulty paying the monthly bills*, *p* = 0.028, *d* = 0.46). In the case of item 22, parent-child arguments were more common during lockdown among families in which a loved one had contracted COVID-19 (*M* = 3.29, *SD* = 1.08 *vs*. *M* = 2.73, *SD* = 1.06), whereas the results for item 25 showed that those families without an affected family member or close friend reported more financial difficulties (*M* = 2.08, *SD* = 1.16 vs. *M* = 1.54, *SD* = 1.09).

Finally, the variable that most influenced foster families’ perceptions of their family context before and during lockdown was having a child with special educational needs at home. It can be seen in Table 5 that this variable was associated with significant differences on eight items for ratings during lockdown, compared with just four items before lockdown. Specifically, when asked to consider the period before lockdown, foster families that *did not* have a child with special educational needs tended to score more positively. This trend was maintained for ratings during lockdown, with the exception of item 21 (*In general, the atmosphere in my family is good*) and item 22 (*Arguments between parents and children*), on which families that *did include* a child with special educational needs scored more positively. The effect sizes for these differences were moderate.

## Discussion

The aim of this study was to assess the impact of the COVID-19 lockdown on the family context of foster and non-foster families, focusing particularly on family cohesion and adaptability and the level of perceived stress. We also examined a series of variables that may have had an influence on foster families’ perceptions of their family context during lockdown.

The results suggest the existence of possible differences between the two types of families, and generally speaking the impact of lockdown seems to have been greater among non-foster families, who perceived a slight improvement in their family functioning (total score on the FACES-20Esp), emotional bonding (cohesion), and in the ability of the family system to adapt its usual rules, power structure, and roles to the new situation brought about by the COVID-19 lockdown (adaptability). Specifically, they reported feeling closer to one another and ascribed greater importance to the family unit as a life support system. This sense of greater closeness appears to derive from their having more ‘quality time’ together in which they could share interests and hobbies, and also from the greater involvement of all family members, especially children, in dealing with problems and challenges. This latter aspect was reflected in a more democratic approach to parenting, one based on dialogue and reasoning. Consistent with these improvements, the level of stress associated with parent-child arguments, child care responsibilities, and a lack of time to relax or switch off was perceived by non-foster families to be lower during lockdown. Overall, this would appear to point to an improved family context, which has been shown to have positive repercussions for the psychological wellbeing of all family members, whether children, adolescents or adults (Prioste et al., 2020; Uruk et al., 2007).

Foster families also perceived an improvement in family functioning (cohesion and adaptability) during lockdown. Specifically, and consistent with the experience of non-foster families, they reported greater involvement of all family members in problem solving, increased mutual support, and more sharing of interests and hobbies.

Analysis of the family context of foster and non-foster families before and during lockdown also suggests some differences in their perceptions, especially as regards the period before lockdown. Specifically, when asked to consider their family functioning prior to lockdown, foster families reported a more positive perception, characterized by greater cohesion and a greater ability to adapt, than did non-foster families. Importantly, this more positive outlook was despite the fact that, in comparison with non-foster families, they also reported more stress associated with financial difficulties and the burden of their children’s school work.

As various studies have indicated, children in foster care are more likely to experience behavior problems and learning difficulties, and also to have special needs in terms of education and mental health, all of which may interfere with their development and academic achievement (Bernedo et al., 2012, 2014; Leloux-Opmeer et al., 2017; McNicholas et al. 2011; Palacios et al., 2014). This places increased stress on foster families, insofar as one of their key roles is to support the child through his or her schooling (Mendis et al., 2015; Zetlin et al., 2010). Accordingly, we found here that foster families who had a child with special educational needs reported higher levels of stress associated with the burden of school work, and this was the case both before and during lockdown.

Interestingly, differences between the two types of families in their perceptions of family context appear to be less marked when they were asked to consider their experiences during lockdown. Nevertheless, foster families continued to perceive stronger emotional bonding and greater involvement of children in resolving problems and in decision making over suitable punishment for their behavior. It is possible that the more positive perceptions that foster families had of their family context both before and during lockdown are the result of the training that they will have previously received through child protection agencies, which is a legal requirement for prospective foster families in Spain (Law 1/1996, subsequently modified by Law 26/2015).

We also found that differences between the two types of families in perceived stress related to paying the monthly bills disappeared when they reported on their experience during lockdown, as compared with previously. Some families suffered loss of employment, and hence greater financial difficulties, as a result of the COVID-19 lockdown (Moya et al., 2020), and this could account for why non-foster families came to experience similar levels of stress to foster families. Furthermore, the level of financial stress experienced by foster families may have been buffered to some extent by the monthly allowance that, under Spanish law (Directive of July 26, 2017), they receive for each foster child. Whatever the case, it should be noted that the overall stress level reported by foster families was lower during (as compared with before) lockdown, whereas that of non-foster families remained more or less the same.

A related and important point to consider is that although COVID-19 poses a risk to the whole of society, the impact of the pandemic and the resulting lockdown on specific families and individuals is shaped by socioeconomic differences (Moya et al., 2020). The results of this study appear to support this, insofar as those foster families that did not experience loss of employment during lockdown seemed to have a more positive perception of their family context, characterized by a greater ability to adapt, fewer financial difficulties, and stronger family cohesion. These findings are consistent with the work of Estévez (2016), who argues that stress related to financial hardship undermines parents’ competencies and increases the likelihood that they will adopt a parenting style that is less sensitive to their child’s needs.

With respect to those foster families where a relative or close friend had contracted COVID-19, the results showed that they reported higher levels of stress related to parent-child arguments and financial difficulties. Importantly, having a child with special educational needs posed an additional challenge for foster families. This is consistent with the results of a study by Brown and Rodger (2009), who found that families who had fostered a child with functional diversity reported difficulties with managing multiple roles and dealing with the child’s challenging behavioral challenges, in addition to the costs associated with fostering a child with a disability and problems related to obtaining specialized health and educational services.

In summary, despite the presence of certain factors that may have a negative impact on family context, the COVID-19 lockdown appears to have provided many families with an opportunity to reappraise their family system and to strengthen their emotional bonding and capacity to adapt. Research suggests that greater family cohesion and adaptability can lead to improved psychological adjustment among family members (Pollock et al., 2014; Prioste et al., 2020). Likewise, the use of more democratic parenting styles can help children to develop their social skills, sense of identity, and self-esteem, and in the specific case of foster children it can reduce the stress they experience when placed with a new family (Jiménez and Palacios, 2009; Salas et al., 2015, 2016).

In order to address those risk factors that, according to our findings and those of previous studies, may cause stress to families and have a negative impact on family functioning, we would urge authorities to implement the Council of Europe’s (2006) Recommendation (Rec2006/19) on Policy to Support Positive Parenting. The primary aim in doing so would be to strengthen families’ competencies and ensure they have the support and resources they need to fulfill their responsibilities. Initiatives of this kind, and especially advice on parenting, are now recognized to be of vital importance for children, families, and society as a whole (Bornstein, 2002; Rodrigo et al., 2015). A guiding principle of the positive parenting perspective in this respect is that although all families, and especially those who foster a child, are likely to experience periods of stress and risk, a strengths-based approach is the best way of promoting and supporting their functioning (Julien-Chinn et al., 2017; Lietz et al., 2016).

### Limitations

The main limitation of this study concerns the use of retrospective measures of pre-pandemic family functioning, given that participants may have inaccurate or distorted recall (Curtiss et al., 2000). In this regard, longitudinal studies would be useful to determine the extent to which the perceived effects of lockdown are maintained over time. Another limitation is that data were collected through an online survey, which means that people without access to the internet or who are not regular users were either excluded or likely to be missed as potential respondents. In addition, we deliberately used a relatively small number of questionnaire items so as to avoid fatigue among participants, but this may have biased the results by overlooking aspects of importance to families’ experience of lockdown. A related limitation concerns the small convenience sample of foster families. It is important to recognize, however, that recruiting these families can be difficult, because it is first necessary to contact foster care agencies. Furthermore, their availability may depend on whether or not they are already participants in other research projects.

### Implications for Practice

Notwithstanding the aforementioned limitations, our findings suggest that lockdown has been perceived by many families as an opportunity to improve their family context. Given that further lockdowns of some degree may need to be implemented in the future, it is essential to ensure that families have access to resources that will help them cope with the experience, for example, programs aimed at developing their communication and conflict resolution skills, and measures that help them reconcile work and family life. These resources will need to be adapted to the specific needs of different types of families, hence the importance of the present analysis of foster and non-foster families. Our results suggest that in order to cope with situations such as the COVID-19 lockdown, families need to pay particular attention to their style of communication, emotional bonding, and ability to redefine family rules and norms. They should also be encouraged to sign up for positive parenting courses.
